# Nontarget Catches of Green and Brown Lacewings (Insecta: Neuroptera: Chrysopidae, Hemerobiidae) Collected by Light- and Volatile-Baited Traps in the Transcarpathian Lowland (W Ukraine)

**DOI:** 10.3390/insects16010074

**Published:** 2025-01-14

**Authors:** Kálmán Szanyi, Antal Nagy, Aletta Ősz, Levente Ábrahám, Attila Molnár, Miklós Tóth, Szabolcs Szanyi

**Affiliations:** 1 Faculty of Agriculture and Food Sciences and Environmental Management, Institute of Plant Protection, University of Debrecen, H-4032 Debrecen, Hungary; szanyi.kalman@agr.unideb.hu (K.S.); nagyanti@agr.unideb.hu (A.N.); 2 For the Nature- and Environmental Protection—PAPILIO (NGO), UA-89463 Velyka Dobron’, Ukraine; 3 Plant Protection Institute, Centre for Agricultural Research, Hungarian Research Network, H-1022 Budapest, Hungary; alikkafox@gmail.com (A.Ő.); toth.miklos@atk.hun-ren.hu (M.T.); 4 Rippl-Rónai Museum, H-7400 Kaposvár, Hungary; labraham@smmi.hu; 5 Department of Zoology and Ecology, Hungarian University of Agriculture and Life Sciences, H-2100 Gödöllő, Hungary; athoeska@gmail.com

**Keywords:** *Cunctochrysa albolineata*, lures, phenylacetaldehyde, isoamyl alcohol, chemical ecology

## Abstract

Distribution data on the lacewing fauna of the data-deficient Transcarpathian Lowland (West Ukraine) were provided. The attractivity of phenylacetaldehyde-(FLO) and isoamyl alcohol-based (SBL) lures designed for trapping lepidopteran pests to lacewings was also studied and compared to the efficiency of light traps traditionally used in studies on neuropterans. In the three-year study, 374 individuals of 10 species were caught. Although the light trap was the most efficient method, the efficiency of the tested lures could also be proved. Regarding abundances, FLO was significantly more efficient than the SBL lure. The lures could supplement the checklist of the fauna with two species and attracted an especially high number of *Chrysoperla* species. In the case of parallel use with light traps, they serve as an efficient standardised combined method for trapping lacewings, both in faunistic studies and plant protection applications.

## Simple Summary

Plant volatile traps designed for catching Lepidoptera pests also captured a high number of lacewings, allowing us to study their odour preferences and compare their attractivity to the traditionally used light trap method. The results of this study suggest that the phenylacetaldehyde- and isoamyl alcohol-based volatile baits can effectively supplement traditional light traps for surveying lacewings. Additionally, this study provides the first data on the mass attraction of the species *Cunctochrysa albolineata* to volatile lures.

## 1. Introduction

The lacewing fauna of Ukraine is relatively well studied—e.g., 30 species of Chrysopidae and 34 species of Hemerobiidae are known in the country—but there are notable spatial differences in the intensity of the studies and available distribution data [1,2,3,4,5,6]. While the fauna of mountainous areas, such as the Carpathians, is well known [1,4,6,7], fewer data are available from lowland regions, including the Transcarpathian Lowland [6]. However, the latter forms a biogeographically transitional zone between the Pannonian and Carpathian regions, resulting in a highly diverse and unique wildlife population [8,9].

Lacewings, especially *Chrysoperla* species, respond to various volatile compounds emitted by plants or synthetic and semi-synthetic mixtures. Methyl salicylate (MeSA) is found to attract the species *Chrysopa nigricornis* Burmeister, 1839, and *Chrysoperla carnea* Complex [10]. The attractivity of the compound is higher for *Chr. carnea* Complex mixed with phenylacetaldehyde and acetic acid [11]. Aphid sex pheromones like nepetalactol and nepetalactone attract male lacewings, but floral baits negatively affect their effectiveness [12]. Additionally, squalene attracts both *Chrysoperla* and *Chrysopa* species [13]. Thus, synthetic and semi-synthetic volatiles can efficiently attract lacewings, increasing their role in biological plant protection.

The primary aim of this study is to provide distribution data on the lacewing fauna of the Transcarpathian Lowland, a data-deficient region in western Ukraine. This study also tests the effectiveness of two volatile lures designed for plant protection monitoring of lepidopteran pests [14,15] for lacewing sampling and attraction, and compares their efficiency to the traditionally used light trap method.

## 2. Materials and Methods

Field investigations were carried out at the margin of the Velyka Dobron’ Game Reserve (Transcarpathia, West Ukraine; GPS: N48.4424°, E22.4076°). The sampling site is located in the Bereg Plain, situated on the border between the moderately warm and moderately cool climate zones (annual average temperature: 9.5–9.6 °C; summer average temperature: 16.8–16.9 °C). The area is moderately dry, with a total annual precipitation of 610 to 630 mm, of which 360 to 370 mm occurs during the growing season [16]. The area is mainly dominated by hardwood gallery forests, oak–hornbeam forests, silver lime–oak forests, forest fringes, tall herb communities, humid clearings, and willow scrubs [17]. The sampling site is marginated with agricultural fields.

Samplings were conducted from 1 June to 19 October 2014, from 24 May to 11 October 2015, and from 10 April to 11 September 2016, covering the phenology of the adults of most green and brown lacewing species. CSALOMON^®^ VARL+ funnel traps (Plant Protection Institute, CAR, Budapest, Hungary) were placed on the trees at a forest edge, at a height of 1.8–2.0 m with 20 m distances; photos of the trap can be viewed at www.csalomontraps.com (accessed on 10 December 2024). Synthetic phenylacetaldehyde-based (FLO) and semi-synthetic isoamyl alcohol-based lures (SBL) were used in the funnel traps which are developed for catching and monitoring lepidopteran pests [14,15]. Since the tested volatiles are not species-specific, they attracted a wide range of non-target taxa in addition to the targeted Lepidoptera pests. The collected material included many non-target Lepidoptera [18], Diptera [19], Orthoptera [17], and Neuroptera species. 

The semi-synthetic bait (SBL) is a mixture of isoamyl alcohol, acetic acid, and red wine (1:1:1), imitating the scent of tree sap, and potentially attracts species inhabiting arboreal habitats [20]. FLO lure contains floral scent compounds—a mixture of phenylacetaldehyde, eugenol, and benzyl acetate (1:1:1)—and mainly attracts flower-visiting insects [20]. Synthetic compounds (>95% purity) applied in baits were obtained from Sigma-Aldrich Kft. (Budapest, Hungary).

Traps containing the tested volatiles (SBL, FLO) and the unbaited control traps (UNB) were used in four repetitions. The traps were checked and rotated weekly to reduce the bias of the location. Vaportape^®^ II pesticide strips were used as a killing agent.

In 2015, a Jermy-type light trap was installed near the volatile traps, operating with a 120 W mercury-vapour lamp.

The green and brown lacewings caught were identified to the species level based on the keys of [21,22].

To evaluate the attractivity of the tested baits, we compared the total number and mean number of individuals (individuals/trap/sample). Data normality and variance homogeneity were tested with Q-Q plots and the Levene test, respectively. As these assumptions were not met, the non-parametric Kruskal–Wallis test was used. Where significant differences were found, treatments were compared using the Mann–Whitney U-test. Statistical analyses were performed with SPSS 21.0 software [23].

## 3. Results

During the three-year study, 374 specimens of 10 Neuroptera species were collected (Table 1). Eight of these species belong to the Chrysopidae family, while the other two belong to the Hemerobiidae family. The most abundant genus was *Chrysoperla*, followed by *Cunctochrysa*. Accordingly, the *Chrysoperla carnea* Complex dominated the assemblage of the area (RF% = 54.50), followed by *Cunctochrysa albolineata* (Killington, 1935) (RF% = 20.11) and *Chrysopidia ciliata* (Wesmael, 1841) (RF% = 11.90). The relative frequencies of the other seven species were under 14%.

**Figure 1:**
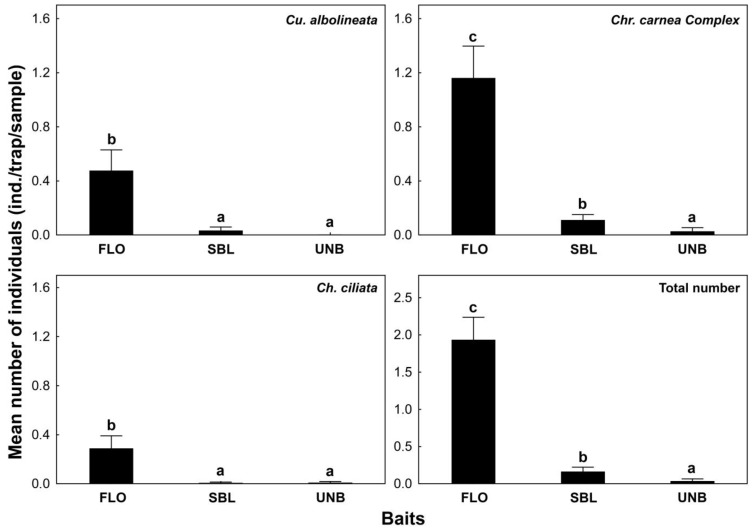
Number of caught lacewings (individuals/trap/sample) attracted by different baits. Letters show significant differences based on the Mann–Whitney U-test (*p* < 0.05).

Based on the three-year data, significant differences were found in the efficiency of traps baited with different lures (Figure 1). Traps baited with phenylacetaldehyde- (FLO) and isoamyl alcohol-based lures (SBL) attracted significantly more lacewing individuals compared to the unbaited control traps. Additionally, the attractivity of the FLO lure was significantly higher than that of the SBL lure.

Both volatiles attracted four species—*Chrysoperla carnea* Complex, *Chrysopidia ciliata, Cunctochrysa albolineata,* and *Apertochrysa prasina*, but only the abundance of *Cu. albolineata, Chr. carnea* Complex, and *Ch. ciliata* were high enough for statistical analysis. All three species were attracted by FLO lure significantly higher than by the SBL lure or the unbaited control. However, the efficiency of the SBL bait was only higher than the UNB’s in the case of *Chr. carnea* Complex (Figure 1).

**Figure 2:**
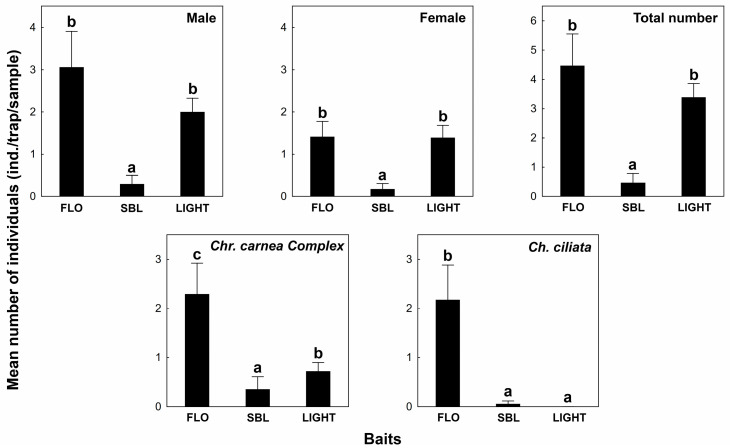
Mean number of caught lacewings (individuals/trap/sample, by species and by sexes) attracted by different baits in 2015. Letters show significant differences based on the Mann–Whitney U-test (*p* < 0.05).

In 2015, traps baited with FLO lure caught lacewings with a higher number than the light trap, but the difference was not significant (Figure 2). Both the FLO lure and the light attracted lacewings with significantly higher numbers than the SBL lure. The bait preferences of the sexes were similar, but the males showed higher attractivity to all the tested lures (FLO, SBL, Light).

The abundance of *Chrysoperla carnea* Complex and *Chrysopidia ciliata* were high enough for statistical analysis. The attractivity of FLO lure was significantly higher than the other baits in the case of both species. For *Ch. ciliata,* differences were not found between the attractivity of the SBL lure and the light trap, but *Chr. carnea* Complex was significantly more attracted by the light trap.

Traps baited with the FLO lure caught more lacewings, but the light trap attracted more species. *Chrysopa perla*, *C. nigricostata*, *C. phyllochroma*, *C. walkeri*, *Hemerobius humulinus*, and *Micromus variegatus* were caught exclusively by the light trap, while the differential species of the volatile lures were only the *Ch. ciliata* and *Apertochrysa prasina*.

## 4. Discussion

This study provides data on the lacewing fauna of the data-deficient Transcarpathian Lowland (W Ukraine). The presence of 10 species was recorded in the area, belonging to the families of Chrysopidae and Hemerobiidae, with the absolute dominance of *Chrysoperla carnea* Complex, common in agricultural habitats [24,25].

The results confirm the efficiency of the light trapping method for faunistic studies of lacewings [26,27], attracting a wider range of species. However, species *Chrysopidia ciliata* and *Apertochrysa prasina* were caught only with volatile baited traps, suggesting that the synthetic and semi-synthetic baits used can supplement the traditionally used light trap.

The phenylacetaldehyde-based lure (FLO) was more efficient in attracting *Chrysoperla* and *Chrysopidia* species (*Chr. carnea* Complex and *Ch. ciliata*), proving the results of previous studies on the attraction of *Chrysoperla* species by volatiles [10,11,13]. The attractivity of isoamyl alcohol-based lure (SBL) was higher than the control only in the case of *Chr. carnea* Complex, but traps baited with volatiles (FLO and/or SBL separately) caught the species *Cunctochrysa albolineata* and *Ap. prasina*, providing the first data on the mass attraction of the species *Cu. albolineata* to volatile lures [28].

Although light trapping still seems to be the most efficient method for surveying lacewing assemblages, phenylacetaldehyde- and isoamyl alcohol-based baits may provide additional data, catching some differential species that cannot be sampled with the traditional methods. This provides further opportunities in both faunistic investigations and biological plant protection.

## Figures and Tables

**Table 1:** Number of caught lacewing species and individuals by trap type and in total across the three years of sampling in the Velyka Dobron’ Game Reserve (Transcarpathian Lowland, West Ukraine). Sdiff = differential species caught only with a given trap type.

Species	2014			2015				2016			SUM
	FLO	SBL	UNB	FLO	SBL	Light	UNB	FLO	SBL	UNB	
*Chrysopa perla* (Linnaeus, 1758)	0	0	0	0	0	14	0	0	0	0	14
*Chrysopa nigricostata* (Brauer, 1850)	0	0	0	0	0	2	0	0	0	0	2
*Chrysopa phyllochroma* Wesmael, 1841	0	0	0	0	0	3	0	0	0	0	3
*Chrysopa walkeri* (McLachlan, 1893)	0	0	0	0	0	17	0	0	0	0	17
*Chrysoperla carnea* Complex	22	6	0	39	6	13	3	112	5	0	206
*Chrysopidia ciliata* Wesmael, 1841	0	0	0	37	1	0	1	6	0	0	45
*Cunctochrysa albolineata* Killington, 1935	71	5	0	0	0	0	0	0	0	0	76
*Apertochrysa prasina* Burmeister, 1839	1	1	0	0	1	0	0	0	0	0	3
*Hemerobius humulinus* Linnaeus, 1758	0	0	0	0	0	2	0	0	0	0	2
*Micromus variegatus* (Fabricius, 1793)	0	0	0	0	0	10	0	0	0	0	10
Total number of individuals	94	12	0	76	8	30	4	118	5	0	378
Total number of species	3	3	0	2	3	7	2	2	1	0	10
Sdiff	0	0	0	0	1	6	0	1	0	0	-

## Data Availability

The datasets generated during and/or analysed during the current study are available from the corresponding author on reasonable request.

## References

[1] Seredyuk G.V. (2013). Neuropterous (Insecta: Neuroptera) Primeval Beech Forests Uholka Array CBR. Sci. Bull. Uzhhorod Univ. Ser. Biol..

[2] Seredyuk G.V. (2015). Green Lacewings (Insecta: Neuroptera, Chrysopidae) Fauna of Ukraine. Proc. State Nat. Hist. Mus..

[3] Seredyuk G.V. (2016). Neuropterida (Insecta: Neuroptera) of Ukrainian Carpathians. Ukr. Entomol. J..

[4] Seredyuk G.V. (2016). Neuropterous Subfamily Nothochrysinae (Insecta: Neuroptera, Chrysopidae) Fauna of Ukraine. Proc. State Nat. Hist. Mus..

[5] Seredyuk G.V. (2019). Insecta Neuroptera of the Galician National Park. Proc. State Nat. Hist. Mus..

[6] Seredyuk G.V. (2020). Altitude and Biotope Distribution of Species of a Number of Neuroptera Fauna of the Ukrainian Carpathians and the Transcarpathian Lowlands. Proc. State Nat. Hist. Mus..

[7] Biodiversity of Ukraine—The Information Resource for Ukraine Biota Diversity. State Museum of Natural History, National Academy of Science of Ukraine. http://dc.smnh.org/.

[8] Varga Z. (1995). Geographical Patterns of Biodiversity in the Palearctic and in the Carpathian Basin. Acta Zool. Acad. Sci. Hung..

[9] Varga Z., Láng I., Bedő Z., Csete L. (2003). The Zoogeography of the Carpathian Basin. Növény, Állat, Élőhely.

[10] James D.G. (2003). Field Evaluation of Herbivore-Induced Plant Volatiles as Attractants for Beneficial Insects: Methyl Salicylate and the Green Lacewing, *Chrysopa nigricornis*. J. Chem. Ecol..

[11] Tóth M., Szentkirályi F., Vuts J., Letardi A., Tabilio M.R., Jaastad G., Knudsen G. (2009). Optimization of a Phenylacetaldehyde-Based Attractant for Common Green Lacewings (*Chrysoperla carnea* s.l.). J. Chem. Ecol..

[12] Koczor S., Szentkirályi F., Pickett J., Birkett M., Tóth M. (2015). Aphid Sex Pheromone Compounds Interfere with Attraction of Common Green Lacewings to Floral Bait. J. Chem. Ecol..

[13] Koczor S., Szentkirályi F., Tóth M. (2019). New Perspectives for Simultaneous Attraction of *Chrysoperla* and *Chrysopa* Lacewing Species for Enhanced Biological Control. Sci. Rep..

[14] Szanyi S., Attila M.-G., Lajos K., Tímea S., Zoltán V., Miklós T., Antal N. (2019). Nyírségi Macroheterocera Együttesek Vizsgálata Illatanyagcsapdák Alkalmazásával. Erdészettudományi Közlemények.

[15] Nagy A., Szarukán I., Szalárdi T., Szanyi S., Jósvai J.K., Tóth M. (2022). Addition of 4-Oxoisophorone Improves Performance of Bisexual Lure for *Autographa gamma* (L.) (Lepidoptera: Noctuidae). J. Appl. Entomol..

[16] Dövényi Z. (2010). Inventory of Microregions in Hungary.

[17] Nagy A., Ősz A., Tóth M., Rácz I.A., Kovács S., Szanyi S. (2023). Nontarget Catches of Traps with Chemical Lures May Refer to the Flower-Visitation, Probable Pollination, and Feeding of Bush Crickets (Ensifera: Tettigoniidae). Ecol. Evol..

[18] Nagy A., Szarukán I., Gém F., Nyitrai R., Füsti-Molnár B., Némerth A., Kozák L., Molnár A., Katona K., Szanyi S. (2015). Preliminary Data on the Effect of Semi-Synthetic Baits for Noctuidae (Lepidoptera) on the Non-Target Lepidoptera Species. Acta Agrar. Debreceniensis.

[19] Nagy A., Katona P., Molnár A., Rádai Z., Tóth M., Szanyi K., Szanyi S. (2023). Wide Range of Brachyceran Fly Taxa Attracted to Synthetic and Semi-Synthetic Generic Noctuid Lures and the Description of New Attractants for Sciomyzidae and Heleomyzidae Families. Insects.

[20] Tóth M., Szarukán I., Nagy A., Gém F., Nyitrai R., Kecskés Z., Krakkó L., Jósvai J.K., Bélai I. (2015). Félszintetikus “Biszex” Csalétkek Kártevő Rovarok Nőstényeinek és Hímjeinek Fogására. Növényvédelem.

[21] Aspöck H., Aspöck U., Hölzel H. (1980). Die Neuropteren Europas. Eine Zusammenfassende Darstellung der Systematik, Ökologie Und Chorologie der Neuropteroidea (Megaloptera, Raphidioptera, Planipennia) Europas.

[22] Thierry D., Cloupeau R., Jarry M., Canard M. (1998). Discrimination of the West-Palearctic *Chrysoperla* Steinmann species of the *carnea* Stephens group by means of claw morphology (Neuroptera, Chrysopidae). Acta Zool. Fenn..

[23] Ketskeméty L., Izsó L., Könyves Tóth E. (2011). Bevezetés az IBM SPSS Statistics Programrendszerbe.

[24] Mahzoum A.M., Villa M., Benhadi-Marín J., Pereira J.A. (2020). Functional Response of *Chrysoperla carnea* (Neuroptera: Chrysopidae) Larvae on *Saissetia oleae* (Olivier) (Hemiptera: Coccidae): Implications for Biological Control. Agronomy.

[25] Ranjbar Aghdam H., Nemati Z. (2020). Modeling of the Effect of Temperature on Developmental Rate of Common Green Lacewing, *Chrysoperla carnea* (Steph.) (Neuroptera: Chrysopidae). J. Biol. Pest Control.

[26] Nabli H., Bailey W.C., Necibi S. (1999). Beneficial Insect Attraction to Light Traps with Different Wavelengths. Biol. Control.

[27] Deutsch B., Paulian J.A., Thierry D., Canard M. (2005). Quantifying Biodiversity in Ecosystems with Green Lacewing Assemblages. Agron. Sustain. Dev..

[28] Koczor S., Szentkirályi F., Birkett M.A., Pickett J.A., Voigt E., Tóth M. (2010). Attraction of *Chrysoperla carnea* Complex and *Chrysopa spp.* Lacewings (Neuroptera: Chrysopidae) to Aphid Sex Pheromone Components and a Synthetic Blend of Floral Compounds in Hungary. Pest Manag. Sci..

